# KRAB zinc finger protein diversification drives mammalian interindividual methylation variability

**DOI:** 10.1073/pnas.2017053117

**Published:** 2020-11-25

**Authors:** Tessa M. Bertozzi, Jessica L. Elmer, Todd S. Macfarlan, Anne C. Ferguson-Smith

**Affiliations:** ^a^Department of Genetics, University of Cambridge, CB2 3EH Cambridge, United Kingdom;; ^b^The Eunice Kennedy Shriver National Institute of Child Health and Human Development, The National Institutes of Health, Bethesda, MD 20892

**Keywords:** DNA methylation, endogenous retrovirus, KRAB zinc finger proteins, metastable epiallele, VM-IAP

## Abstract

Most transposable elements (TEs) in the mouse genome are heavily modified by DNA methylation and repressive histone modifications. However, a subset of TEs exhibit variable methylation levels in genetically identical individuals, and this is associated with epigenetically conferred phenotypic differences, environmental adaptability, and transgenerational epigenetic inheritance. The evolutionary origins and molecular mechanisms underlying interindividual epigenetic variability remain unknown. Using a repertoire of murine variably methylated intracisternal A-particle (VM-IAP) epialleles as a model, we demonstrate that variable DNA methylation states at TEs are highly susceptible to genetic background effects. Taking a classical genetics approach coupled with genome-wide analysis, we harness these effects and identify a cluster of KRAB zinc finger protein (KZFP) genes that modifies VM-IAPs in *trans* in a sequence-specific manner. Deletion of the cluster results in decreased DNA methylation levels and altered histone modifications at the targeted VM-IAPs. In some cases, these effects are accompanied by dysregulation of neighboring genes. We find that VM-IAPs cluster together phylogenetically and that this is linked to differential KZFP binding, suggestive of an ongoing evolutionary arms race between TEs and this large family of epigenetic regulators. These findings indicate that KZFP divergence and concomitant evolution of DNA binding capabilities are mechanistically linked to methylation variability in mammals, with implications for phenotypic variation and putative paradigms of mammalian epigenetic inheritance.

## Significance

Transposable elements (TEs) are repetitive sequences with potential to mobilize, causing genetic diversity. To restrict this, most TEs in the mouse are heavily epigenetically modified by DNA methylation. However, a few TEs exhibit variable methylation levels that differ between individuals and confer an epigenetic, rather than genetic, influence on phenotype. The mechanism underlying this remains unknown. We report the identification of a polymorphic cluster of KRAB zinc finger proteins (KZFPs) responsible for the epigenetic properties of these variably methylated TEs, with deletion of the cluster profoundly influencing their DNA methylation and expression of adjacent genes. We propose that rapid KZFP divergence underlies variable epigenetic states in mammals, with implications for epigenetically conferred phenotypic differences between individuals within and across generations.

Complex genetic interactions contribute to evolutionary fitness, phenotypic variation, and disease risk. This is highlighted by comparative research across inbred mouse strains showing that genetic background not only influences basic fitness traits such as litter size and sperm count but also modulates the penetrance and expressivity of numerous gene mutations (1, 2). Despite the extensive documentation of strain-specific epistatic effects in the mouse and their important implications for mechanistic insight and experimental reproducibility, the underlying modifier genes remain uncharacterized in most cases.

Studies on foreign DNA insertions in the mouse genome demonstrate that modifier genes can act via epigenetic pathways to drive genetic background–dependent phenotypes. A number of transgenes show predictable strain-specific DNA methylation patterns that are associated with transgene expression levels (3–6). Similar effects have been reported on the methylation state of endogenous retroviruses (ERVs), as exemplified by the MusD ERV insertion *Dac*^*1J*^, which is methylated in mouse strains that carry the unlinked *Mdac* modifier gene (7, 8). In strains lacking the *Mdac* allele, *Dac*^*1J*^ is unmethylated and the mice exhibit limb malformation.

Another example is provided by the *Agouti viable yellow* (*A*^*vy*^) metastable epiallele, in which a spontaneously inserted intracisternal A-particle (IAP) element influences the expression of the downstream coat-color gene *Agouti* (9). IAPs are an evolutionarily young and highly active class of ERV (10). Variable DNA methylation of the *A*^*vy*^ IAP is established early in development across genetically identical mice and is correlated with a spectrum of coat color phenotypes, which in turn display transgenerational inheritance and environmental sensitivity (11–13). Both the distribution and heritability of *A*^*vy*^ phenotypes are influenced by genetic background (14–16). Therefore, the identification and characterization of the responsible modifier genes can provide insight into the mechanisms governing the early establishment of stochastic methylation states at mammalian transposable elements.

We recently conducted a genome-wide screen for individual variably methylated IAPs (VM-IAPs) in the C57BL/6J (B6) inbred mouse strain (17, 18). The screen yielded a robust set of experimentally validated regions to use as a model to investigate interindividual epigenetic variability. Most VM-IAPs belong to the IAPLTR1_Mm and IAPLTR2_Mm subclasses. Approximately half of them are full-length IAPs with an internal coding region flanked by near-identical long terminal repeats (LTRs); the other half are solo LTRs. While solo LTRs lack autonomous retrotransposition potential, they are rich in regulatory sequences and thus have the ability to affect host gene expression. As observed for *A*^*vy*^, methylation variability is reestablished at VM-IAPs from one generation to the next regardless of parental methylation states. Importantly, VM-IAP methylation levels are consistent across all tissues of a single mouse, suggesting that individual-specific methylation states are acquired in early development prior to tissue differentiation. The interindividual variability suggests that the establishment of VM-IAP methylation levels involves an early stochastic phase.

Here, we introduce genetic variation to the study of VM-IAPs. We report that half of the IAPs found to be variably methylated in B6 are present in 129 substrains, while the vast majority are absent from the CAST/EiJ (CAST) genome. We find that a subset of the shared loci between B6 and 129 display variable methylation in both stains; the remainder are hypermethylated in 129. Further methylation quantification in reciprocal B6 × CAST F1 hybrids reveals pervasive maternal and zygotic genetic background effects. Through backcrossing and genetic mapping experiments, we identify a cluster of KRAB zinc finger proteins (KZFPs) on chromosome 4 responsible for the strain-specific *trans*-acting hypermethylation of multiple B6 VM-IAPs. We show that deletion of the KZFP cluster leads to a decrease in DNA and H3K9 methylation, an increase in H3K4 trimethylation, and alterations in nearby gene expression at the targeted VM-IAPs. A phylogenetic sequence analysis demonstrates that genetic sequence plays a crucial role not only in the targeting of VM-IAPs by strain-specific KZFPs but also in the establishment of methylation variability in a pure B6 context. Based on our findings, we propose that KZFP diversification is at the center of the mechanism leading to variable epigenetic states within and across mouse strains.

## Results

### VM-IAPs Exhibit Strain-Specific Methylation States.

**Fig. 1.**
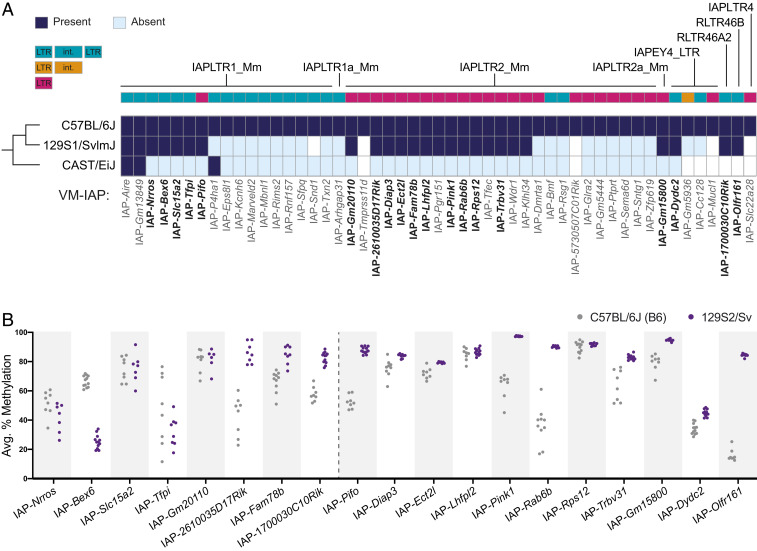
Interindividual methylation variability at IAPs is strain specific. (*A*) B6 VM-IAPs are polymorphic across inbred mouse strains. All experimentally validated B6 VM-IAPs (18) were scored for presence (navy blue rectangles) or absence (light-blue rectangles) in the 129S1/SvlmJ and CAST/EiJ reference genomes. Instances in which a classification could not be made with confidence because of gaps in the reference sequences are shown in white. VM-IAPs are color coded according to their structure (full-length IAPs, blue; truncated IAPs, orange; solo LTRs, pink; key not drawn to scale). LTR subclass annotation, as defined by RepeatMasker, is indicated above each VM-IAP. VM-IAPs are named based on their closest coding gene. (*B*) DNA methylation levels in B6 (gray) and 129S2/Sv (purple) inbred mice of IAPs shared between the two strains. Some IAPs exhibit variable methylation (>10% variance across individuals) in both strains (left of dotted line); others are only variably methylated in B6 mice (right of dotted line). Methylation levels of the distal-most CpGs of the IAP 5′ LTRs were quantified from genomic DNA using bisulphite pyrosequencing. Each dot represents the average methylation level across CpGs for one individual.

To determine whether B6 VM-IAPs are variably methylated in other inbred mouse strains, we first cataloged their presence or absence in the 129S1/SvlmJ (129) and CAST strains based on a previous analysis of polymorphic ERVs (19). The classification was verified, and at times corrected, by visually assessing each locus in the 129 and CAST reference genomes (20). Out of 51 experimentally validated B6 VM-IAPs (18), 25 of the IAPs are present in 129 and 3 are present in CAST (Fig. 1*A*). These numbers are consistent with our previous work showing that VM-IAPs are evolutionarily young IAPs (17) and were expected given the evolutionary relationship between these three strains: B6 and 129 are classical inbred laboratory strains derived from several subspecies, while CAST is wild derived and evolutionarily more distant.

We compared the methylation level of 19 loci conserved between B6 and 129 by bisulphite pyrosequencing. The probed cytosine–guanine dinucleotide (CpG) sites are comparable across loci and located at the most distal end of the 5′ LTR of each element, close enough to the bordering unique sequence to ensure amplification of a single product. As expected, all 19 regions exhibited methylation variability across inbred B6 mice (i.e., more than 10% variability across individuals). In contrast, only eight loci were variably methylated in 129 mice (Fig. 1*B*). Most of these displayed distinct methylation ranges compared to those observed in B6. The remaining 11 IAPs lacked interindividual variability and are therefore not VM-IAPs in the 129 strain (Fig. 1*B*). For the most part, these elements were highly methylated, akin to the vast majority of the ∼10,000 IAPs in the mouse genome. The susceptibility of VM-IAPs to genetic background effects provides an opportunity to map the genetic determinants of interindividual methylation variability.

**Fig. 2.**
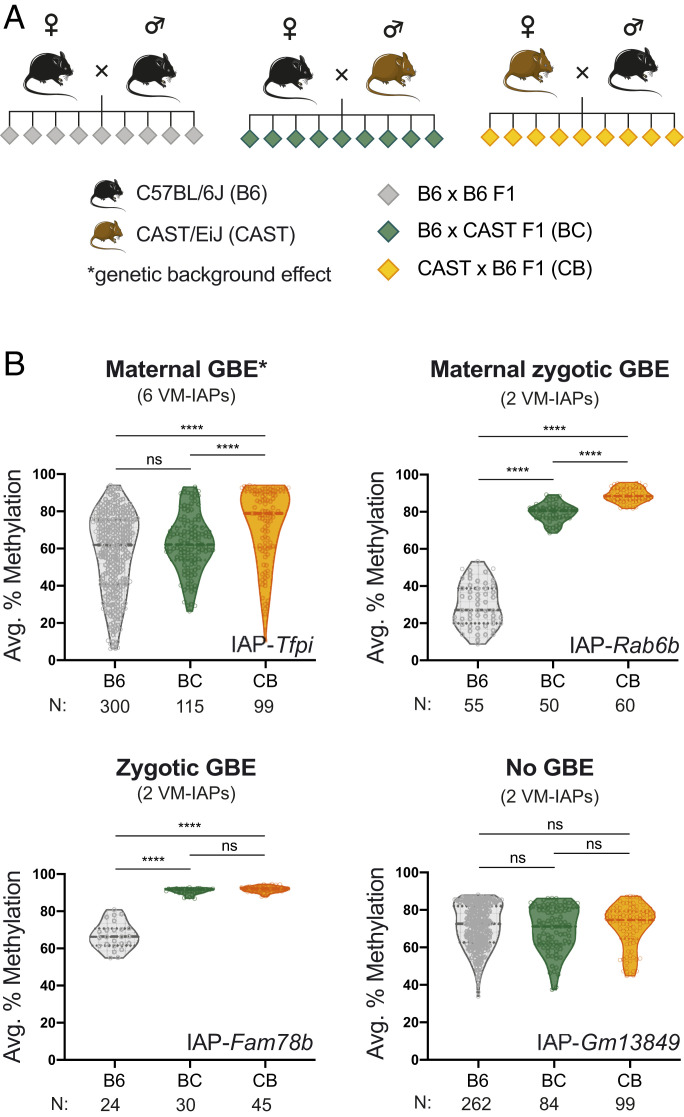
VM-IAP methylation levels are subject to maternal and zygotic genetic background effects (GBEs). (*A*) A reciprocal hybrid breeding scheme. BC F1 hybrids (green diamonds) were generated by breeding B6 (black) females with CAST (brown) males. CB F1 hybrids (yellow diamonds) were produced from the reciprocal cross of CAST females and B6 males. (*B*) VM-IAPs classified based on their susceptibility to maternal GBEs (*Upper Left*), maternal zygotic GBEs (*Upper Right*), zygotic GBEs (*Lower Left*), or neither (*Lower Right*). The violin plots represent the B6 (gray), BC (green), and CB (yellow) F1 offspring methylation distributions. The dotted and dashed lines show the distribution quartiles and median, respectively. The faint hollow circles represent individual-specific methylation levels, quantified from genomic DNA and averaged across the distal CpGs of the VM-IAP 5′ LTR. B6, BC, and CB methylation levels were compared for each VM-IAP using the Kruskal-Wallis test followed by Dunn’s post hoc multiple comparison test. The sample sizes are shown below each graph. Graphs for the eight additional VM-IAPs analyzed in this experiment can be found in *SI Appendix*, Fig. S2. ****P < 0.0001; ns, not significant

Because of the repetitive nature of IAPs, it is difficult to rule out the possibility that the differences in methylation between B6 and 129 are a result of sequence divergence within the elements themselves rather than a consequence of *trans*-acting modifiers. For example, a LINE element is embedded in IAP-*Rab6b* in the 129 genome that is absent in the B6 genome (*SI Appendix*, Fig. S3*A*). To avoid this confounder, we implemented a hybrid breeding scheme using B6 and CAST mice (Fig. 2*A*). Because B6 VM-IAPs are largely absent from the CAST genome, F1 hybrid offspring inherit a single allele from their B6 parent. This property allowed us to assess whether a haploid CAST genome is capable of inducing methylation changes at B6-specific VM-IAPs in *trans*. Maternal and paternal transmission of these alleles was followed by crossing B6 females to CAST males (BC) and CAST females to B6 males (CB), respectively. The reciprocal design enabled the exploration of parent-of-origin effects in addition to genetic background effects for the 12 B6-specific VM-IAPs examined in this experiment. Furthermore, we used large sample sizes to guarantee the detection of subtle shifts in the distribution of methylation levels at each locus, which additionally revealed that the frequency distributions of VM-IAP methylation levels in the pure B6 population form skewed bell curves rather than normal distributions (*SI Appendix*, Fig. S1).

Two-thirds of the assessed B6-specific VM-IAPs showed significant differences between BC and CB methylation distributions (Fig. 2*B* and *SI Appendix*, Fig. S2). These effects were not reciprocal, indicating they were not imprinting effects. For instance, at half of the loci, CB hybrids showed hypermethylation of the paternally inherited B6 VM-IAP, while the BC hybrids exhibited levels comparable to pure B6. This suggests the presence of a CAST-specific maternally inherited modifier acting on the paternally inherited B6 allele (Fig. 2*B* and *SI Appendix*, Fig. S2). Paternal transmission of the CAST modifier had no effect on the maternally inherited B6 VM-IAP, consistent with a maternal effect. Additional experiments are required to better understand these maternal genetic background effects, but strain-specific factors derived from the oocyte are likely involved.

In addition to genetic background–specific maternal effects, a subset of VM-IAPs exhibited zygotic genetic background effects, defined as changes in VM-IAP methylation caused by the introduction of a CAST haploid genome regardless of parental origin. Four VM-IAPs displayed significant shifts in methylation when BC and CB were compared to the B6 population (Fig. 2*B* and *SI Appendix*, Fig. S2). IAP-*Marveld2* was alone in showing a reduction in methylation in F1 hybrids compared to B6 individuals. The other three (IAP-*Rab6b*, IAP-*Sema6d*, and IAP-*Fam78b*) were hypermethylated in F1 hybrids compared to B6 mice, suggesting that CAST-encoded modifiers may be targeting these loci for repression. We note that IAP-*Rab6b* and IAP-*Sema6d* displayed both maternal and zygotic genetic background effects, while IAP-*Gm13849* and IAP-*Slc15a2* displayed neither. The range of responses indicates that the mechanisms influencing variable methylation at IAPs are not common across all loci.

### Strain-Specific IAP-*Rab6b* Hypermethylation Is Driven by a Single-Modifier Locus.

A successful genetic mapping experiment relies on an unambiguous phenotype. Unlike most of the VM-IAPs examined in hybrids, IAP-*Rab6b* (a solo LTR) exhibited nonoverlapping B6 and F1 hybrid methylation distributions that were clearly distinguishable using a 60% methylation threshold (Fig. 2*B*). Because of the categorical nature of this “methylation phenotype,” IAP-*Rab6b* was selected to identify VM-IAP modifiers using B6/CAST hybrids.

**Fig. 3.**
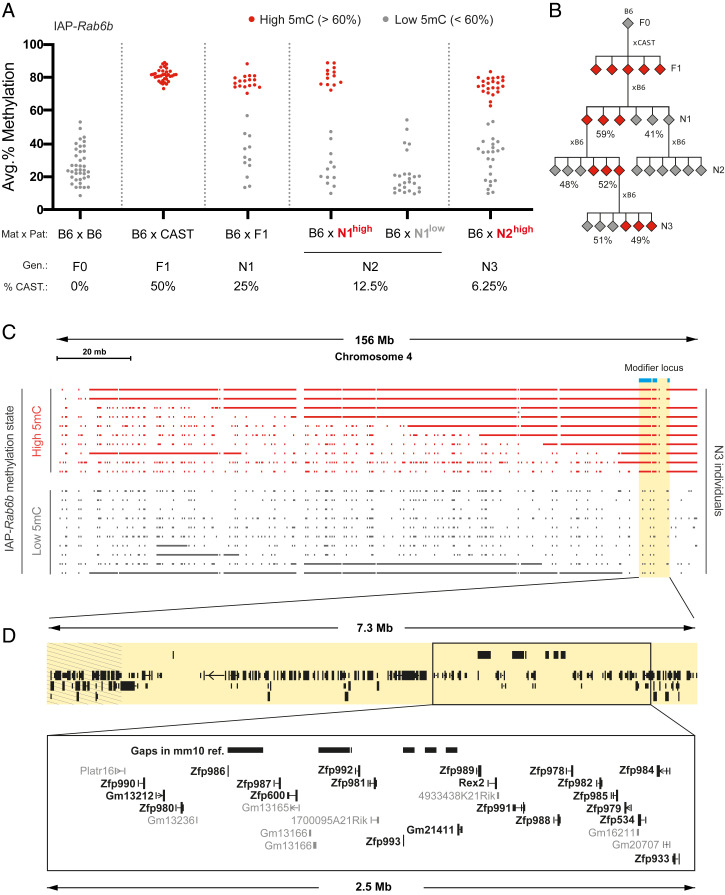
Strain-specific IAP-*Rab6b* hypermethylation is driven by a single dominant modifier locus on chromosome 4. (*A*) Genetic backcrossing uncovers a Mendelian inheritance pattern of IAP-*Rab6b* methylation states. F1 BC males were backcrossed to B6 females to produce the first backcrossed generation (N1). Three highly methylated (red) and three lowly methylated (gray) N1 males were backcrossed to B6 females to produce the N2 generation, and highly methylated N2 males were once again backcrossed to B6 females to produce the N3 generation. The average percent CAST DNA remaining in the genome at each generation is indicated under the graph. A cutoff value of 60% methylation was used to classify individuals as highly (red) or lowly (gray) methylated. (*B*) Pedigree illustrating the inheritance patterns of IAP-*Rab6b* methylation states. The percentages reflect the data in *A*. (*C*) Genetic mapping of the modifier locus to a 7.3-Mb interval on distal chromosome 4 using the GigaMUGA SNP microarray. A map is shown of heterozygous SNPs along the chromosome that are informative between B6 and CAST in 20 N3 individuals (full set of individuals shown in *SI Appendix*, Fig. S4). The heterozygous SNPs shared among all highly methylated N3 individuals and absent from all lowly methylated N3 individuals are shown in blue. The corresponding mapped region is highlighted in yellow. (*D*) An expanded view of the 2.5-Mb KZFP cluster located within the mapped interval. Sequence gaps in the current reference genome (GRCm38/mm10) are displayed as black boxes above the annotated genes. The stripped region represents the portion of DNA excluded by our independent analysis of N2 individuals using the MiniMUGA SNP microarray. The KZFP genes are bolded. Annotations were lifted from the University of California Santa Cruz Gencode V24 track.

We first investigated whether the low methylation state (<60%) could be rescued in a subsequent generation by backcrossing F1 hybrids to B6 mice. Low methylation was reacquired in approximately half of the N1 backcrossed offspring, irrespective of parental origin (Fig. 3*A* and *SI Appendix*, Fig. S3*B*). This is indicative of limited redundancy in IAP-*Rab6b*–targeting modifiers in the CAST genome. The segregation of methylation states was not attributable to the IAP-*Rab6b* copy number, as hemi- and homozygous individuals were represented in both the highly and lowly methylated groups (*SI Appendix*, Fig. S3*C*).

We conducted an additional round of B6 backcrossing to further characterize the inheritance pattern of IAP-*Rab6b* methylation states. N2 offspring generated from highly methylated N1 males recreated the 1:1 ratio of high-to-low methylation observed in N1 offspring, while N2 offspring generated from lowly methylated N1 males were all lowly methylated (Fig. 3 *A* and *B*). This Mendelian inheritance pattern indicates that a single dominant CAST-derived locus causes the hypermethylation of B6-derived IAP-*Rab6b* in *trans*. This was confirmed by crossing highly methylated N2 males to B6 females, once again producing roughly equal numbers of highly and lowly methylated N3 offspring (Fig. 3 *A* and *B*).

We next mapped the modifier locus using the Giga Mouse Universal Genotyping Array (GigaMUGA), a 141,090 single-nucleotide polymorphism (SNP) microarray designed to capture the genetic diversity found across mouse strains (21). Because of the evolutionary distance between B6 and CAST, a majority of the probed SNPs are informative between the two strains. DNA samples from 47 N3 individuals were analyzed on the array. The SNP calls were filtered to identify heterozygous SNPs shared by all 23 highly methylated individuals and absent from all 24 lowly methylated individuals. The resulting SNPs all mapped to a 7.3-Mb interval on distal chromosome 4 (Fig. 3*C*; *SI Appendix*, Fig. S4; and Dataset S1, Table S1). We separately analyzed 22 N2 individuals using the lower-resolution MiniMUGA array and independently identified the same genomic region (Dataset S1, Table S2). Combining both mapping experiments delimited a 6.4-Mb window on chromosome 4 containing the IAP-*Rab6b* modifier(s) (GRC38/mm10, chr4:141964197–148393136). Of note, this locus was found to exhibit high heterozygous SNP density in a study comparing the genomes of sixteen different laboratory mouse strains (20).

Assessment of the genes within the mapped interval revealed a cluster of KZFPs (Fig. 3*D*). Present in the hundreds, KZFPs make up the largest and most diverse transcription factor family in higher vertebrate genomes (22, 23). They are best known for their role in sequence-dependent transposable element repression. Their C_2_H_2_ zinc finger arrays recognize and bind DNA motifs with high specificity, and their KRAB domain recruits the scaffold protein KAP1, which in turn induces heterochromatin (24). The rapid evolutionary expansion of murine KZFPs sets them apart from other more conserved epigenetic regulators. We therefore hypothesized that this KZFP cluster, designated Chr4-cl, contains the strain-specific modifier(s) of IAP-*Rab6b*. The B6, CAST, and 129 variants of this cluster are henceforth referred to as Chr4-cl^B6^, Chr4-cl^CAST^, and Chr4-cl^129^, respectively.

### The CAST-Derived Modifier Locus Targets Multiple VM-IAPs in a Sequence-Specific Manner.

**Fig. 4.**
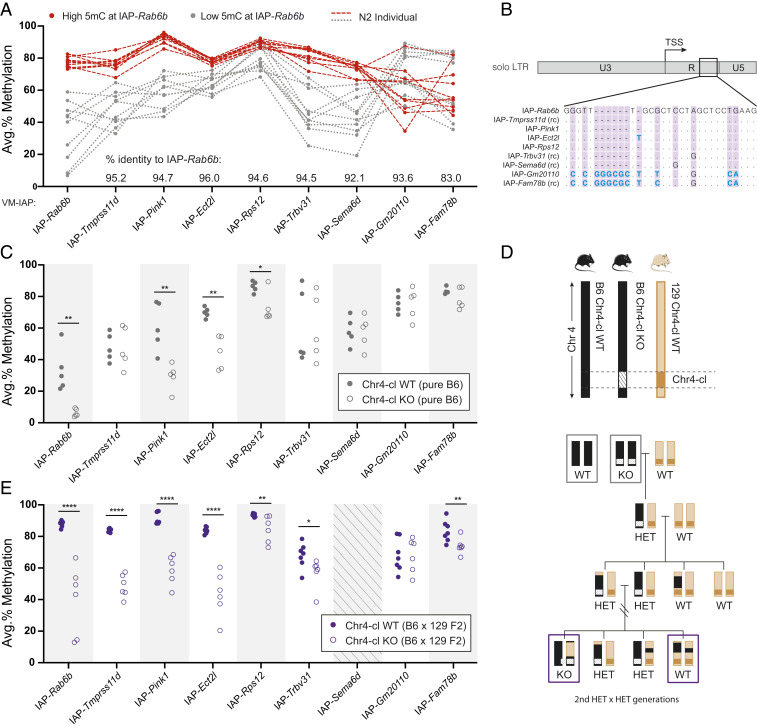
The KZFP cluster Chr4-cl modulates the methylation state of multiple VM-IAPs. (*A*) A cross-locus comparison of N2 methylation states. The methylation levels were quantified at IAP-*Tmprss11d*, IAP-*Pink1*, IAP-*Ect2l*, IAP-*Rps12*, IAP-*Trbv31*, IAP-*Sema6d*, IAP-*Gm20110*, and IAP-*Fam78b* in 20 N2 individuals. The IAP-*Rab6b* methylation level had previously been determined to be high (>60%, red) or low (<60%, gray). The red dashed and gray dotted lines connect the average methylation values of N2 individuals across regions. The percent sequence identity to IAP-*Rab6b*, as determined by the BLAST-like alignment tool (BLAT) (46), is shown for each locus above the x-axis. (*B*) Alignment of VM-IAP LTR sequences in the region displaying divergence between Chr4-cl targets and nontargets. Contraoriented elements were reverse complemented (rc) prior to generating the alignment. The dots represent conserved bases, dashes indicate lack of sequence, and divergent bases are shown in blue. The full-length alignment can be found in *SI Appendix*, Fig. S5. (*C*) Methylation quantification of genomic DNA extracted from Chr4-cl WT mice (gray circles) and Chr4-cl KO mice (hollow gray circles) on a pure B6 genetic background. (*D*) A diagram of the Chr4-cl KO location (Chr4:145383918–147853419, GRCm38/mm10) and breeding scheme used for the data presented in *D* and *E*. The Chr4-cl KO was generated in B6 mice, which were subsequently backcrossed to the 129 × 1/SvJ strain. (*E*) The methylation quantification of genomic DNA extracted from Chr4-cl WT mice (purple circles) and Chr4-cl KO mice (hollow purple circles) on a mixed B6/129 F2 genetic background. IAP-*Sema6d* was excluded from this analysis because it is absent from the 129 genome (Fig. 1*A*). Statistics for *D* and *E*: unpaired *t* tests with false discovery rate of 5% computed using the two-stage step-up method of Benjamini, Krieger, and Yekutieli. *q < 0.05; **q < 0.01; ****q < 0.0001.

IAP sequences are highly repetitive in the mouse genome because of the evolutionary youth and retrotransposition potential of IAP elements (10). In view of the sequence-specificity of KZFP-induced epigenetic repression, we reasoned that VM-IAPs with sequence similarity to IAP-*Rab6b* may also be targeted by the same modifier locus. Six solo LTR VM-IAPs with more than 90% sequence identity to IAP-*Rab6b* were selected as potential targets along with IAP-*Sema6d* and IAP-*Fam78b*, which had exhibited hypermethylation in F1 hybrids (Fig. 2*B*). Methylation was quantified in N2 individuals, half of which were highly methylated at IAP-*Rab6b* (i.e., heterozygous carriers of the CAST modifier locus) and half of which were lowly methylated at IAP-*Rab6b* (i.e., noncarriers of the CAST modifier locus). We found that individuals that were highly methylated at IAP-*Rab6b* were also highly methylated at six out of the eight assessed loci—IAP-*Tmprss11d*, IAP-*Pink1*, IAP-*Rps12*, IAP-*Trbv31*, IAP-*Ect2l*, and IAP-*Sema6d*—and vice versa for the lowly methylated individuals (Fig. 4*A*). This result suggests that these regions are additional modifier targets. In contrast, IAP-*Gm20110* and IAP-*Fam78b* methylation levels were not concordant with IAP-*Rab6b* methylation levels (Fig. 4*A*). A sequence alignment of the nine solo LTR VM-IAPs revealed a single region that distinguished IAP-*Gm20110* and IAP-*Fam78b* from the other six IAPs (Fig. 4*B* and *SI Appendix*, Fig. S5). The 28-base-pair (bp) DNA segment, containing an insertion and various SNPs in IAP-*Gm20110* and IAP-*Fam78b*, is a likely binding site for the CAST-specific modifier.

The cross-locus comparison highlights the sequence dependence of the modifiers of these epialleles and provides support for a KZFP-mediated mechanism. Interestingly, our earlier observations in pure 129 mice showed variable methylation at IAP-*Gm20110* and IAP-*Fam78b* and hypermethylation at the other six IAPs (Fig. 1*B*), suggesting that Chr4-cl^129^ shares VM-IAP modifier allele(s) with Chr4-cl^CAST^ that are absent from Chr4-cl^B6^.

### The KZFP Cluster on Chromosome 4 Modifies VM-IAP Methylation States.

The unique clustered organization of KZFPs in the mouse genome stems from segmental duplications, resulting in high sequence similarity among adjacent KZFPs and low-quality cluster reference sequences (22). To circumvent the technical difficulties and potential functional redundancy associated with generating single-KZFP knockouts (KOs), we examined the consequences of deleting the entire Chr4-cl using a previously generated Chr4-cl KO mouse line (25).

We first assessed DNA methylation effects caused by the loss of Chr4-cl in a pure B6 genetic background. Compared to wild-type (WT) mice, which exhibited the expected interindividual methylation variability at all loci, Chr4-cl KO mice showed significantly lower methylation levels at IAP-*Rab6b*, IAP-*Pink1*, IAP-*Ect2l*, and IAP-*Rps12* (Fig. 4*C*). The effect was particularly pronounced at IAP-*Rab6b*, where all KO mice were completely unmethylated. This result shows that Chr4-cl^B6^ is necessary for the acquisition of variable methylation at IAP-*Rab6b* and reveals an important mechanistic role for KZFPs in the stochastic methylation of retrotransposons. Of note, the other VM-IAPs targeted by the CAST-specific modifier did not show a reduction in methylation in the absence of Chr4-cl^B6^. Given the extensive redundancy displayed by KZFPs in the mouse genome (25), it is possible that the variable methylation observed at these regions in B6 mice is conferred by KZFPs located in other clusters.

We next asked whether Chr4-cl can mediate the strain-specific hypermethylation of VM-IAPs using the 129 Chr4-cl locus. Homozygous B6 Chr4-cl KO mice were crossed to WT 129 mice, which harbor Chr4-cl^129^ as well as most VM-IAPs of interest (Figs. 1*A* and 4*D*). F1 mice were backcrossed to 129 followed by two rounds of heterozygous intercrosses (Fig. 4*D*). VM-IAP methylation was assessed in the resulting Chr4-cl KO and WT mice of mixed B6/129 genetic background. In this instance, all of the predicted Chr4-cl targets from our cross-locus comparison in Fig. 4*A* exhibited significantly lower methylation levels in Chr4-cl KO mice compared to their WT counterparts, often reflecting a return to the variable levels observed at these regions in pure B6 mice (Fig. 4*E*). WT methylation levels were largely consistent with the pure 129 data from Fig. 1*B* despite the use of different 129 substrains. These results indicate that Chr4-cl is the functionally relevant segment of the 6.4-Mb interval identified in our mapping experiment and demonstrate that KZFPs are strain-specific VM-IAP modifiers.

### Loss of Chr4-cl Alters the Chromatin and Transcriptional Landscape near Targeted VM-IAPs.

**Fig. 5.**
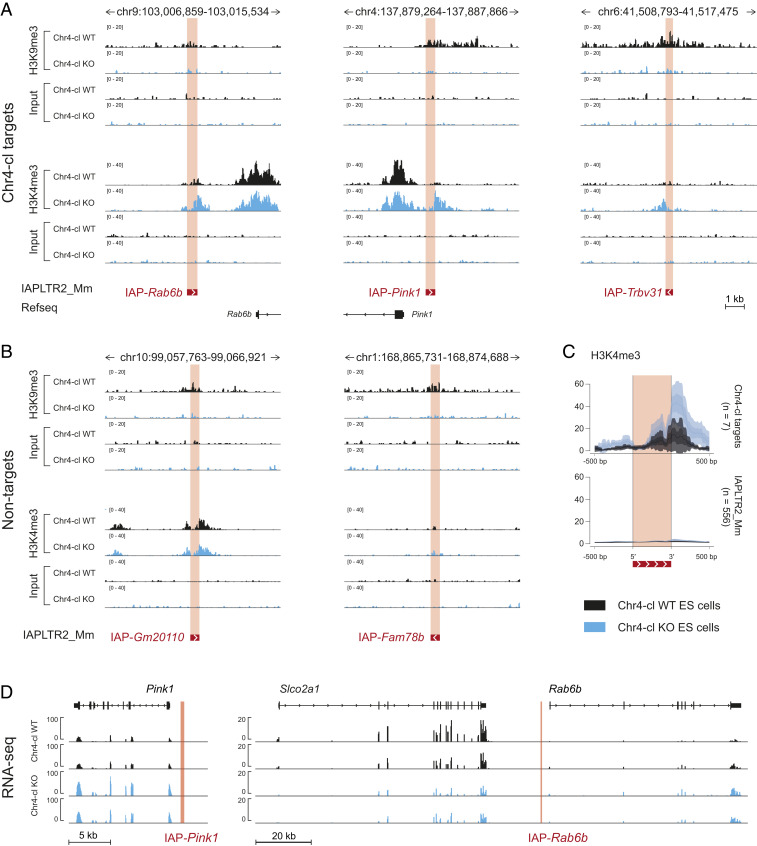
Chr4-cl influences the chromatin and transcriptional landscape at and near targeted VM-IAPs. (*A*) H3K9me3 and H3K4me3 ChIP-seq signal at Chr4-cl target VM-IAPs in Chr4-cl WT (black) and KO (blue) ES cells of mixed B6/129 genetic background. BAM coverage tracks were generated and visualized in IGV. VM-IAPs are shown in red, and directionality is indicated with a white arrow. The NCBI37/mm9 genome coordinates and neighboring annotated genes are displayed above and below the ChIP-seq tracks, respectively. (*B*) As in *A*, but for nontarget VM-IAPs. (*C*) The mean H3K4me3 ChIP-seq signal over the seven confirmed Chr4-cl targets (*Upper*) and over all solo LTRs of the IAPLTR2_Mm subclass in the mouse genome (*Lower*). The dotted lines represent the mean signal, and the shaded regions represent error estimates (SE and 95% CI). Plots were generated using SeqPlots software (47). (*D*) The RNA-sequencing signal from Chr4-cl WT (black) and KO (blue) ES cells of mixed B6/129 genetic background for the genes *Pink1* (upstream of IAP-*Pink1*), *Slco2A1* (upstream of IAP-*Rab6b*), and *Rab6b* (downstream of IAP-*Rab6b*). Two biological replicates per genotype are shown. Datasets were downloaded from the Gene Expression Omnibus database (accession numbers are listed in Dataset S1, Table S6).

The recruitment of KAP1 and subsequent H3K9 trimethylation by the methyltransferase SETDB1 are characteristic of epigenetic silencing by KZFPs. To gain insight into the mechanism by which strain-specific Chr4-cl KZFPs target VM-IAPs, we analyzed previously generated chromatin immunoprecipitation sequencing (ChIP-seq) datasets that profiled histone modifications in Chr4-cl WT and KO embryonic stem (ES) cells of mixed B6/129 background (25). Visual inspection of H3K9me3 ChIP-seq tracks at Chr4-cl–targeted VM-IAPs revealed a modest decrease in H3K9me3 enrichment upon loss of Chr4-cl (Fig. 5*A* and *SI Appendix*, Fig. S6 *A* and *B*). More striking, however, was a marked increase in H3K4me3 in KO cells at Chr4-cl targets, with levels equivalent to those observed at neighboring gene promoters (Fig. 5 *A* and *C* and *SI Appendix*, Fig. S6*A*). No increase in H3K4me3 was observed at nontargets IAP-*Gm20110* and IAP-*Fam78b* (Fig. 5*B*). Therefore, the acquisition of H3K4me3 represents the default chromatin state at these loci, which is partially impeded either directly or indirectly by Chr4-cl KZFPs in early development.

The H3K4me3 mark is associated with transcriptional activity and localizes to gene promoters, with greatest enrichment in the region immediately downstream of the transcription start site (TSS) (26). In line with this, the increase in H3K4me3 in Chr4-cl KO cells at targeted VM-IAPs was exclusively found at their 3′ end, downstream of the TSS embedded in the solo LTRs (Figs. 4*B* and 5 *A* and *C* and *SI Appendix*, Fig. S6*A*).

Next, we explored whether the remodeled chromatin landscape at VM-IAPs in Chr4-cl KO ES cells was associated with the altered expression of neighboring genes. The RNA sequencing (RNA-seq) datasets generated from the same Chr4-cl WT and KO ES cells revealed differences in gene expression near IAP-*Pink1* and IAP-*Rab6b*. *Pink1* and *Rab6b*, located 1 kb upstream and 3 kb downstream of IAP-*Pink1* and IAP-*Rab6b*, respectively, were up-regulated in Chr4-cl KO ES cells (but only *Pink1* reached statistical significance) (Fig. 5*D* and *SI Appendix*, Fig. S6*C*). *Slco2a1*, located 20 kb upstream of IAP-*Rab6b*, was significantly down-regulated in Chr4-cl KO ES cells. These data indicate that the loss of Chr4-cl results in a range of transcriptional disruptions. While transcriptional changes were not observed near the other Chr4-cl–targeted VM-IAPs, our analysis does not rule out longer-range transcriptional effects.

### Methylation Variability at IAPLTR2_Mm Elements Is Sequence Dependent.

**Fig. 6.**
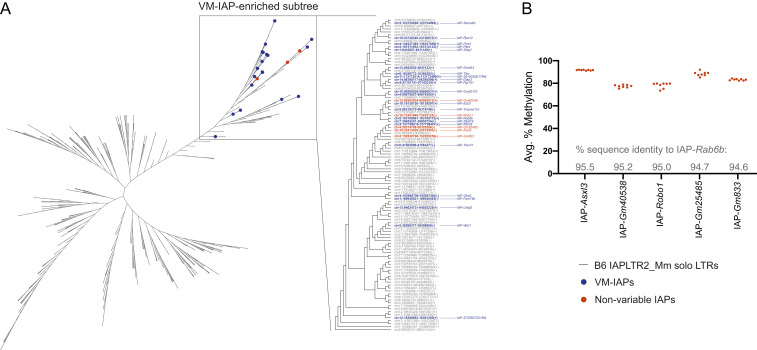
The methylation variability at IAPLTR2_Mm elements is sequence driven. (*A*) A neighbor-joining tree of all solo LTR IAPs of the IAPLTR2_Mm subclass in the B6 genome between 200 and 800 bp in length. The solo LTR sequences were aligned using MUSCLE software, and the neighbor-joining tree was built using Geneious Prime software. The navy blue and orange nodes represent experimentally validated VM-IAPs and nonvariable IAPs, respectively. The VM-IAP–enriched subtree, containing all known IAPLTR2_Mm VM-IAPs (navy), is shown in greater resolution and labeled with GRC38/mm10 genomic coordinates and strandedness. (*B*) The methylation quantification of genomic DNA from eight B6 individuals at five solo LTRs in the VM-IAP–enriched subtree (orange). The percent sequence identity to IAP-*Rab6b* is shown above the x-axis for each IAP.

**Fig. 7.**
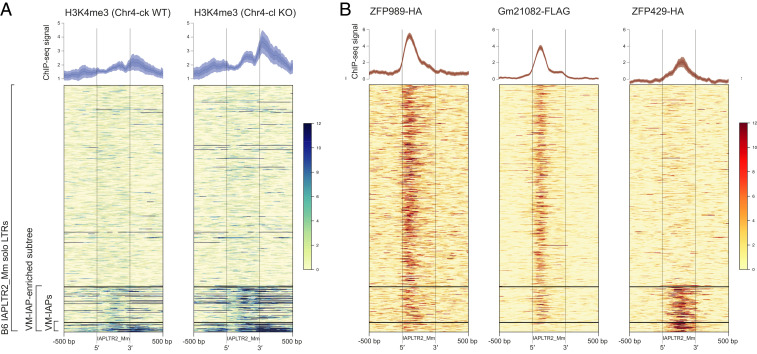
The VM-IAP–enriched subtree exhibits H3K4 trimethylation and distinct KZFP binding. (*A*) Heatmaps of H3K4me3 ChIP-seq coverage in Chr4-cl WT (*Left*) and KO (*Right*) ES cells of mixed B6/129 genetic background over all solo LTRs of the IAPLTR2_Mm subclass (*n* = 556). VM-IAPs and IAPs belonging to the VM-IAP–enriched subtree were clustered for the analysis. All solo LTRs are anchored from their 5′ start to their 3′ end, with a pseudolength of 500 bp. The analysis was extended to 500 bp up- and downstream of each element. The average read coverage is plotted above each heatmap. The dotted lines represent the mean signal, and the shaded regions represent error estimates (SE and 95% CI). The plots and heatmaps were created using SeqPlots (47). (*B*) Heatmaps of overexpressed ZFP989-HA (*Left*), Gm21082-FLAG (*Middle*), and ZFP429-HA (*Right*) ChIP-seq coverage in F9 EC cells over all solo LTRs of the IAPLTR2_Mm subclass (*n* = 556). The plotting settings were as in *A*.

The seven VM-IAPs that we identified as Chr4-cl targets are all solo LTRs of the IAPLTR2_Mm subclass. To determine how VM-IAPs compare to other solo LTRs of this subclass from an evolutionary perspective, we built a neighbor-joining tree of all solo IAPLTR2_Mm elements in the B6 genome. Consistent with a KZFP-mediated mechanism, we found that VM-IAPs of this subclass cluster together phylogenetically (Fig. 6*A*). This is in agreement with our previous analysis on IAPs of the IAPLTR1_Mm subclass (17) and reinforces the concept that genetic sequence is instructive in the establishment of interindividual methylation variability. We selected five epigenetically uncharacterized IAPs in the VM-IAP–enriched subtree to test whether members of this clade are in fact unidentified VM-IAPs. All five candidates failed to display methylation variability, highlighting that other determinants such as genomic context likely also play a role in the acquisition of methylation variability at IAPs (Fig. 6*B*). Given that murine IAPs are almost invariably highly methylated, it follows that the VM-IAP–enriched subtree has escaped epigenetic repression, at least partially. Notably, this clade was enriched in H3K4me3 in WT ES cells and showed a greater increase in H3K4me3 in Chr4-cl KO ES cells compared to other IAPLTR2_Mm solo LTRs (Fig. 7*A*).

### Chr4-cl KZFPs Target IAPLTR2_Mm Elements.

To determine whether Chr4-cl KZFPs are capable of recognizing IAPLTR2_Mm elements, the IAPLTR2_Mm consensus sequence was queried for binding motifs previously assigned to 16 Chr4-cl KZFPs (25). Two of the query hits, ZFP989 and Gm21082, exhibited ChIP-seq enrichment at IAPLTR2_Mm solo LTRs (Fig. 7*B*). Both ZFP989 and Gm21082 appear to bind the same region of the LTR and are thus likely the product of a gene duplication event within Chr4-cl. Gm21082 was previously reported to target IAPLTR2_Mm elements along with one other KZFP, ZFP429, which is located in a KZFP cluster on chromosome 13 (Chr13-cl) (25). Interestingly, ZFP989 and Gm21082 showed reduced enrichment at VM-IAPs compared to other IAPLTR2_Mm solo LTRs, whereas ZFP429 exhibited strong preferential binding at IAPs in the VM-IAP–enriched subtree (Fig. 7*B*).

It is feasible that the CAST and 129 alleles of ZFP989 or Gm21082 are responsible for the strain-specific hypermethylation of VM-IAPs, while ZFP429 may be involved in the establishment of interindividual methylation variability in B6 mice. This would explain why DNA methylation at some of the VM-IAPs targeted by Chr4-cl^129^ were unaffected by the loss of Chr4-cl in a pure B6 background (Fig. 4*C*). We note that while we have shown that multiple KZFPs are capable of binding LTRs of the IAPLTR2_Mm subtype, none of the candidate Chr4-cl KZFPs appear to recognize the predicted binding site identified in Fig. 4*E*. This is unsurprising considering that the B6 alleles of the Chr4-cl KZFP modifiers are not expected to strongly bind VM-IAPs, and the ChIP-seq datasets used for this analysis were generated through stable expression of epitope-tagged B6 KZFPs.

## Discussion

Variable methylation of murine IAPs across genetically identical individuals was reported more than two decades ago (27), yet the underlying mechanisms and evolutionary origins of this phenomenon have remained elusive. In this study, we identify widespread genetic background–specific modification of VM-IAPs and exploit these to investigate the genetic determinants of mammalian epigenetic stochasticity. We demonstrate that a polymorphic KZFP cluster on chromosome 4 promotes the sequence- and strain-specific hypermethylation of multiple VM-IAPs in *trans*, the loss of which alters the chromatin and transcriptional landscape of the modified loci and their surrounding genetic environment. We expect our classical genetics approach using inbred mouse strains to be generalizable to other variably methylated regions in the mouse genome.

The identification of Chr4-cl KZFPs as strain-specific VM-IAP modifiers is consistent with the literature documenting KZFPs as products of rapidly evolving genes with critical functions in transposable element repression (reviewed in ref. 24). The large number of species-specific KZFPs in the mouse compared to most other higher vertebrates suggests that murine KZFPs have undergone particularly rapid amplification (23). It is thought that this expansion reflects an active evolutionary arms race following ERV invasion events (22, 28). Our finding that Chr4-cl contains strain-specific modifiers of certain IAP elements indicates that murine KZFPs are evolving rapidly enough to detect significant divergence within the mouse species with important epigenetic and transcriptional ramifications. IAPs, which are murine specific and represent the most mutagenic ERV class in the mouse (10), have likely played a major role in this process. Thus, comparative research across mouse strains is uniquely suited to the study of KZFP gene evolution.

A significant technical hindrance in taking full advantage of cross-strain mouse genetics in this context relates to the extensive redundancy exhibited by KZFPs both within and across clusters (22, 23, 25). The current mouse strain reference genomes have large gaps in KZFP clusters, rendering a cross-strain comparison of Chr4-cl sequences currently unfeasible (20). In fact, while we expect significant KZFP sequence differences in Chr4-cl across mouse strains, we acknowledge that we have not excluded the possibility that the strain-specific effects we have identified are driven by differences in KZFP gene regulation rather than allelic variation of KZFPs themselves. Indeed, a recent study showed that the NOD and B6 mouse strains exhibit differential T cell gene expression and three-dimensional chromatin organization in Chr4-cl, with implications for diabetes phenotypes (29). The improvement of genetic engineering tools for repetitive gene families and the generation of high-quality mouse strain reference genomes with full coverage over KZFP clusters will be crucial in addressing this issue.

We have shown that in addition to mediating the strain-specific hypermethylation of VM-IAPs, KZFPs play an important role in the establishment of interindividual methylation variability in a pure B6 background, as evidenced by the complete loss of DNA methylation at IAP-*Rab6b* in B6 Chr4-cl KO mice. The specificity of KZFP binding relies on four amino acids within each zinc finger, so mutations in these key residues or in the DNA sequence of their binding sites have important implications for target site binding kinetics (30). We propose that stochastic methylation arises when VM-IAP sequences are weakly recognized by KZFPs during early preimplantation development. Consistent with this model, a large number of murine KZFPs are highly expressed in ES cells, including most of the KZFPs in Chr4-cl (22, 23, 25, 31). Furthermore, we previously documented a lack of methylation covariation across VM-IAPs within an individual mouse (17), which is in agreement with low-affinity binding interactions occurring independently between a KZFP and multiple VM-IAP targets. The phylogenetic clustering of VM-IAPs provides further support for this mechanism given that KZFP function is driven by DNA sequence recognition. It is noteworthy that ZFP429 preferentially binds solo LTRs in the VM-IAP–enriched subtree compared to other IAPLTR2_Mm solo LTRs, perhaps reflecting a host adaptive response to elements that have escaped epigenetic repression. Alternatively, it is possible that interindividual methylation variability is an early sign of transposable element domestication.

While this framework predicts that the sequence of an IAP and that of its KZFP modifiers are the prime drivers of interindividual methylation variability, additional factors are expected to influence the probability of a binding event occurring. This is illustrated by the presence of highly methylated IAPLTR2_Mm elements within the VM-IAP–enriched subtree. Potential influencing factors include chromatin accessibility of VM-IAP insertion sites and the number and expression level of KZFPs targeting a particular locus. Importantly, we envisage that other IAP-binding proteins interfere with binding kinetics between KZFPs and VM-IAPs. In fact, VM-IAPs are enriched for CCCTC-binding factor (CTCF), a methylation-sensitive DNA binding protein that may act as an antagonist to methylation-promoting KZFP modifiers (17, 18).

The most prominent difference in chromatin structure that we observed between Chr4-cl WT and KO ES cells was a substantial increase in H3K4me3 in Chr4-cl KO cells at the 3′ end of targeted VM-IAPs. This suggests that KZFP binding in early development prevents the accumulation of H3K4me3 at VM-IAP TSSs, potentially enabling subsequent DNA methylation. In line with this, the ADD domain of de novo DNA methyltransferases DNMT3A and DNMT3B (and of their cofactor DNMT3L) specifically binds unmethylated H3K4 (32, 33). In the absence of modifying KZFPs, other transcription factors and histone-modifying enzymes have increased access to VM-IAP sequences, which may in turn contribute to the dysregulation of VM-IAP–neighboring genes. Interestingly, a recent study using the BXD recombinant inbred mouse panel identified six major *trans*-acting dominant suppressors of H3K4me3 in male germ cells, all of which were mapped to KZFP clusters (34).

Our work is consistent with previous research on strain-specific modifiers (35). A growing number of ERV-derived mouse mutations whose phenotypic penetrance is dependent on genetic background have been associated with modifier genes located in an interval on Chr13-cl. This region harbors *Mdac,* modifier of the dactylaplasia-causing *Dac*^*1J*^ insertion, as well as modifiers of IAPs shown to mediate a range of strain-specific phenotypes (8, 36–38). Moreover, two polymorphic KZFPs in Chr13-cl, SNERV1 and SNERV2, were recently found to influence ERV expression in lupus-susceptible mouse strains (39). Interestingly, the Chr4-cl KZFP *Zfp979* is responsible for the strain-specific methylation of the HRD transgene (40), indicating that polymorphic Chr4-cl KZFPs target a variety of foreign DNA sequences. Notably, the genes whose expression we found to be altered in Chr4-cl KO mice near Chr4-cl–targeted VM-IAPs have been implicated in human disease. Mutations in *PINK1*, which codes for a kinase involved in mitochondrial quality control, are associated with Parkinson’s disease (41); mutations in *SLCO2A1*, a prostaglandin transporter, cause chronic enteropathy (42). It is possible that Chr4-cl KZFP diversification influences related susceptibilities in mice. Taken together, a picture emerges of KZFPs as fine-tuners of evolution within the mouse species, whereby KZFP divergence across strains leads to strain-specific epigenetic landscapes with important phenotypic implications. Moreover, it is possible that KZFP divergence across human populations contributes to variable phenotypic outcomes via the differential recognition of endogenous (and perhaps even exogenous) retroviruses.

We have shown that most VM-IAPs are susceptible to maternal effects and posit that these, too, are driven by strain-specific KZFPs. A number of KZFPs have been characterized as maternal-effect genes expressed in the oocyte (43–45). Maternally derived ZFP57, for example, is required for the maintenance of DNA methylation at imprinted regions during global postfertilization methylation erasure (43). It is intriguing that, on a B6 background, *A*^*vy*^ and IAP-*Gm13849* only exhibit epigenetic inheritance upon maternal transmission (12, 17). We speculate that certain paradigms regarded as mammalian transgenerational epigenetic inheritance may in actual fact be the product of postfertilization retargeting of epigenetic states by germline-derived KZFPs.

## Materials and Methods

All mouse work was conducted in compliance with the Animals (Scientific Procedures) Act 1986 Amendment Regulations 2012 following ethical review by the University of Cambridge Animal Welfare and Ethical Review Body (Home Office project license number PC213320E). DNA methylation was quantified using bisulphite pyrosequencing, and genetic mapping was carried out using the GigaMUGA SNP genotyping array (21). Data availability and details of mouse experiments, molecular techniques, and computational analyses performed in this study are described in *SI Appendix*, *Materials and Methods*.

## Supplementary Material

Supplementary File

Supplementary File

Supplementary File

Supplementary File

## Data Availability

All study data are included in the article and supporting information.
