# Supplementary material for: KRAB zinc finger protein diversification drives mammalian interindividual methylation variability
Source: Proc Natl Acad Sci U S A. 2020 Nov 25;117(49):31290–300. doi: 10.1073/pnas.2017053117 (PMC7733849; doi:10.1073/pnas.2017053117)
Supplement: Supplementary File [file pnas.2017053117.sapp.pdf]

PNAS
www.pnas.org

Supplementary Information for

KRAB zinc finger protein diversification drives mammalian interindividual methylation variability

Tessa M. Bertozzi, Jessica L. Elmer, Todd S. MacFarlan, Anne C. Ferguson-Smith*

*Email: afsmith@gen.cam.ac.uk

This PDF file includes:

Materials and Methods
Figures S1 to S5
Legends for Datasets S1 to S3
SI References

Other supplementary materials for this manuscript include the following:

Datasets S1 to S3

www.pnas.org/cgi/doi/10.1073/pnas.2017053117

Materials and Methods

Mice. All mouse work was carried out in compliance with the Animals (Scientific Procedures) Act 1986 Amendment Regulations 2012 following ethical review by the University of Cambridge Animal Welfare and Ethical Review Body (Home Office project license # PC213320E). C57BL/6J, 129S2/Sv, and CAST/EiJ mice were obtained from Charles River, Envigo, and the MRC Harwell Institute, respectively. The Chr4-cl KO mouse line was generated by injecting Chr4-cl KO B6 ES cells into B6 blastocysts as previously described (1). Chr4-cl KO/KO mice were bred with 129X1/SvJ mice to produce Chr4-cl KO/+ B6/129 F1 mice, which were subsequently crossed to 129X1/SvJ. Heterozygous intercrosses of the resulting offspring generated the Chr4-cl KO and WT individuals of mixed B6/129 genetic background used in this study. Mice were kept in a temperature- and humidity-controlled environment under a 12h light-dark cycle and were fed standard chow *ad libitum*.

Polymorphism analysis. The presence or absence of VM-IAPs in the 129S1/SvImJ and CAST/EiJ genomes was determined based on a catalogue of transposable element variants across inbred mouse strains (2). The classification was confirmed or corrected by querying the most recent genome assemblies for sequences adjacent to VM-IAP insertions (3). Genomic coordinates for all assessed IAP elements are listed in Dataset S1: Table S3.

DNA extraction. Genomic DNA was extracted from ear or tail tissue. Tissues were digested overnight with Proteinase K and DNA was purified using a standard phenol-chloroform extraction and ethanol precipitation protocol.

IAP-*Rab6b* copy number analysis. IAP-*Rab6b* copy number was determined via PCR amplification. Genomic DNA from N1 individuals was used as template and primers were designed to target the unique regions flanking IAP-*Rab6b* such that the B6 and CAST alleles would amplify 700 and 100 bp fragments, respectively (primer sequences in Dataset S1: Table S4). PCR product sizes were assessed by agarose gel electrophoresis.

Bisulphite pyrosequencing. 1 μg of genomic DNA was bisulphite converted using the Imprint DNA Modification Kit (Sigma) following the two-step modification procedure. Regions of interest were amplified from bisulphite-modified DNA via PCR using biotinylated forward or reverse primers. Amplicons were designed such that either the forward or reverse primer annealed to the unique DNA bordering each repeat element. Primer sequences are listed in Dataset S1: Table S4. PCRs were carried out in triplicate using HotStarTaq DNA Polymerase (QIAGEN) under the following conditions: 95°C x 5 min; 94°C x 30 sec, 56°C x 30 sec, 72°C x 55 sec, 40 cycles; 72°C x 5 min. PCR products were bound to Streptavidin Sepharose High Performance beads (GE Healthcare) by shaking in binding buffer (10mM Tris-HCL pH7.6, 2M NaCl, 1mM EDTA, 0.1% Tween-20) at room temperature. Biotinylated strands were sequentially purified in 70% ethanol, denaturation solution (0.2M NaOH), and wash buffer (10mM Tris-acetate, pH 7.6) using

the PyroMark Q96 Vacuum Workstation (QIAGEN). Sequencing primers were annealed to the purified DNA at 85°C in annealing buffer (20mM Tris-acetate pH7.6, 2mM magnesium acetate) before loading onto the PyroMark Q96 MD pyrosequencer (QIAGEN). Pyrosequencing assays were designed using PyroMark Assay Design SW 2.0 software (QIAGEN) and carried out using PyroMark Gold Q96 Reagents and HS Capillary Tips (QIAGEN) as per the manufacturer's instructions. CpG-site-specific percent methylation (ratio of C-to-T) was calculated using Pyro Q-CpG 1.0.9 software (Biotage). Four to five CpGs were assayed at the distal end of the 5′ LTR of each VM-IAP and their methylation levels were averaged prior to subsequent analysis.

Backcrossing and genetic mapping. B6 x CAST (BC) F1 hybrid males were backcrossed to B6 females to produce the N1 generation. IAP-*Rab6b* methylation levels were quantified in N1 offspring and classified as highly- or lowly methylated using a 60% methylation threshold. Three N1 males of each group were backcrossed to B6 females to generate the N2 generation. The same process produced N3 mice born to highly methylated N2 males. Methylation levels at IAP-*Rab6b* for all individuals used in this experiment were quantified from ear notch samples collected from pups at postnatal day 10 (P10). Genetic mapping was carried out using the MiniMUGA and GigaMUGA (4) SNP genotyping arrays developed at the University of North Carolina at Chapel Hill on the Illumina Infinium platform and operated by Neogen Inc. (Lincoln, NE). 10,819 and 143,090 biallelic SNPs tiled along the mouse genome are interrogated on each array, respectively, most of which are informative between B6 and CAST. 22 P10 N2 individuals (9 highly methylated and 13 lowly methylated) and 47 P10 N3 individuals (23 highly methylated and 24 lowly methylated) were analysed on the MiniMUGA and GigaMUGA, respectively. Ear notches collected from P10 pups were sent to Neogen Inc. for DNA extraction and microarray hybridization. Unprocessed SNP calls are listed in Dataset S2 (MiniMUGA) and Dataset S3 (GigaMUGA). The R package dplyr (5) was used to process and filter genotype calls to identify heterozygous SNPs shared by all highly methylated individuals and absent from all lowly methylated individuals. Heterozygous SNPs that did not match the B6 and CAST consensus SNPs were removed from the analysis.

IAPLTR2_Mm sequence analysis. IAPLTR2_Mm sequences as defined by RepeatMasker were extracted from UCSC using the table browser function and filtered to exclude sequences belonging to full-length IAPs. Solo LTR sequences between 200 and 800 bp in length were aligned using MUSCLE software and an unrooted neighbour-joining tree was constructed using Geneious Prime software.

Analysis of ChIP-seq and RNA-seq datasets. ChIP-seq reads were downloaded from the GEO database in fastq format and mapped to the NCBI37/mm9 reference genome using Bowtie (-5 3 --best). RNA-seq datasets were downloaded in Bigwig format. Bam coverage files and Bigwig files were visualised in the Integrative Genomics Viewer (6), and modified in Adobe Illustrator CC 2020 v24.0. H3K9me3 and H3K4me3 ChIP-seq

experiments as well as RNA-seq experiments were carried out in Chr4-cl WT and KO ES cells of mixed B6/129 genetic background. KZFP ChIP-seq datasets were generated following the overexpression of epitope-tagged Chr4-cl KZFPs in F9 EC cells. All enrichment plots and heat maps were generated using Seqplots (7). Differential gene expression was analysed as previously described (1).

Identification of IAPLTR2_Mm-binding KZFPs. Chr4-cl KZFP binding motifs were identified based on ChIP-seq peaks using MEME as previously described (1). Chr4-cl KZFPs are listed in Dataset S1: Table S5. The IAPLTR2_Mm consensus sequence was extracted from the Dfam database (8) and queried for KZFP binding sites using FIMO (9). Motifs with q-value < 0.05 were selected for further investigation.

Statistical analyses. All statistical tests were conducted as indicated in the appropriate figure legend using GraphPad Prism 8 software.

Data availability. GEO accession numbers for all datasets analysed in this study are listed in Dataset S1: Table S6. Unprocessed MiniMUGA and GigaMUGA SNP calls are listed in Dataset S2 and Dataset S3, respectively.

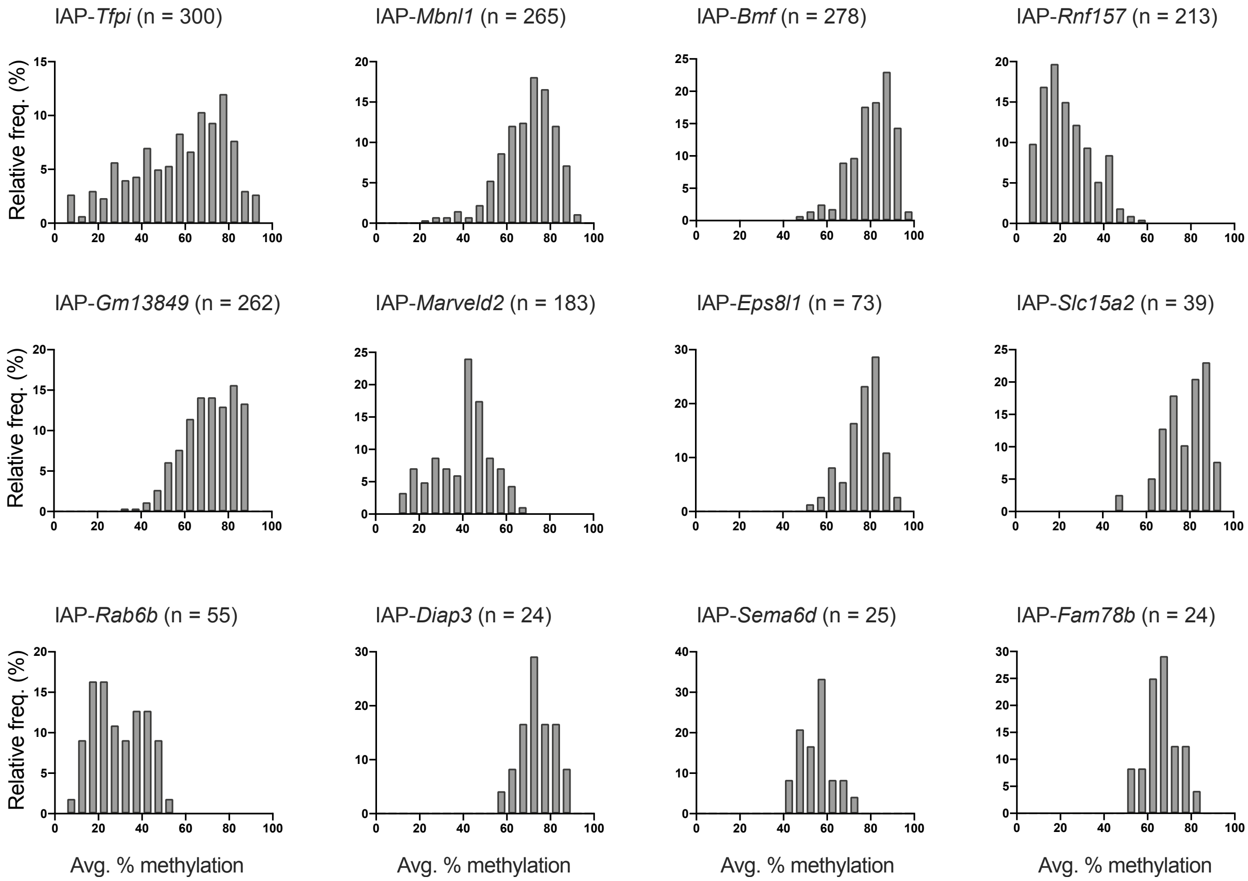

Fig. S1. Frequency distribution histograms of VM-IAP methylation levels in the B6 population. Percent methylation values were averaged across CpGs at each locus and frequency distributions were generated by tabulating the relative frequency of values into 20 bins. Bin width was set to 5%. Sample sizes reflect the number of mice tested for each VM-IAP.

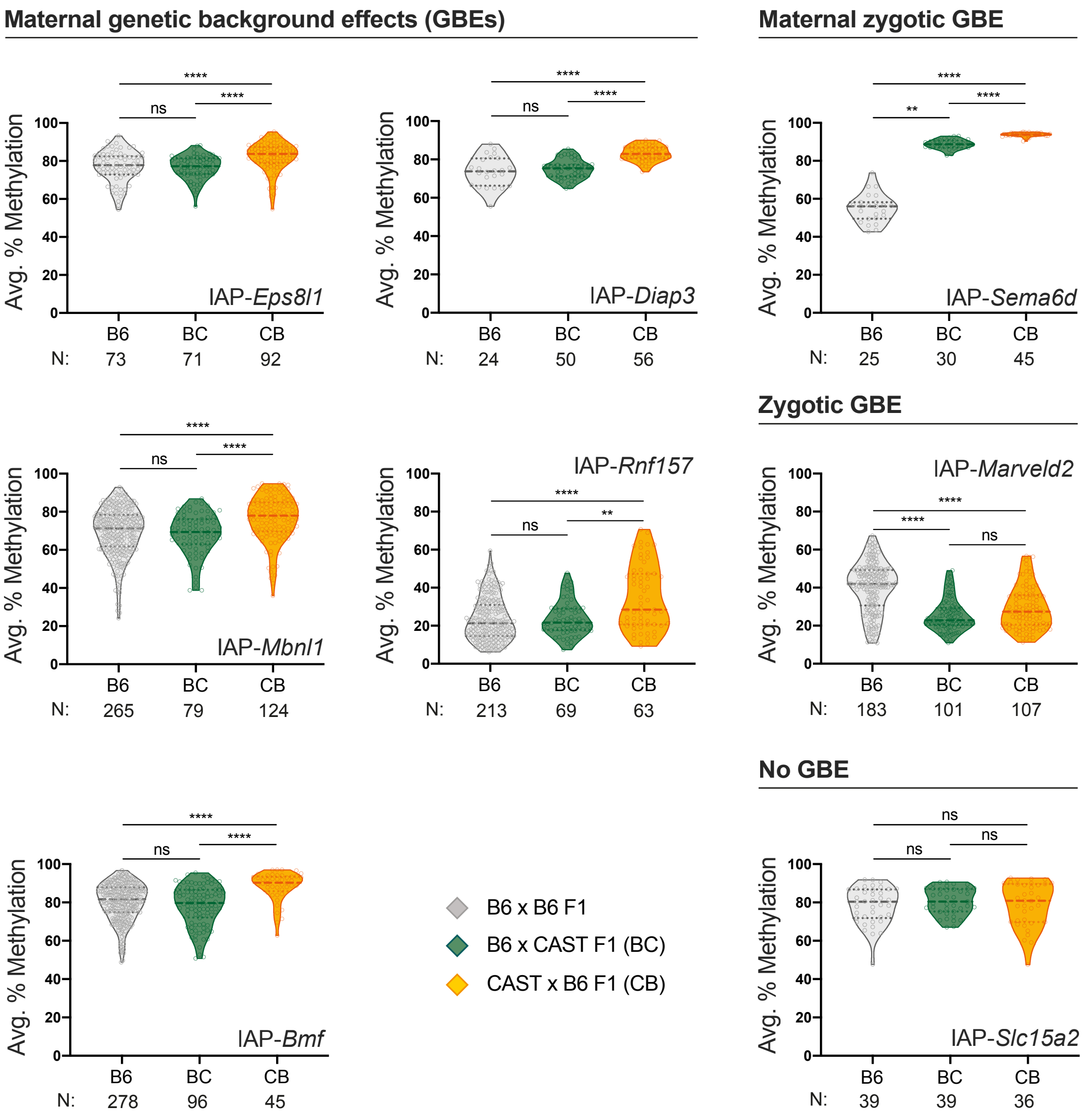

Fig. S2. VM-IAP methylation levels are subject to maternal and zygotic genetic background effects (GBEs). BC F1 hybrids (green diamonds) were generated by breeding B6 females with CAST males. CB F1 hybrids (yellow diamonds) were produced from the reciprocal cross of CAST females and B6 males. VM-IAPs are classified based on their susceptibility to maternal GBEs (left), maternal zygotic GBEs (upper right), zygotic GBEs (middle right), or neither (lower right). Violin plots represent the B6, BC, and CB F1 offspring methylation distributions. Dotted and dashed lines show the distribution quartiles and median, respectively. Faint hollow circles represent individual-specific methylation levels, quantified from genomic DNA and averaged across the distal CpGs of the VM-IAP 5′ LTR. B6, BC, and CB methylation levels were compared for each VM-IAP using the Kruskal-Wallis test followed by Dunn's post hoc multiple comparison test (** $p < 0.01$; **** $p<0.0001$; ns: not significant). Sample sizes are shown below each graph.

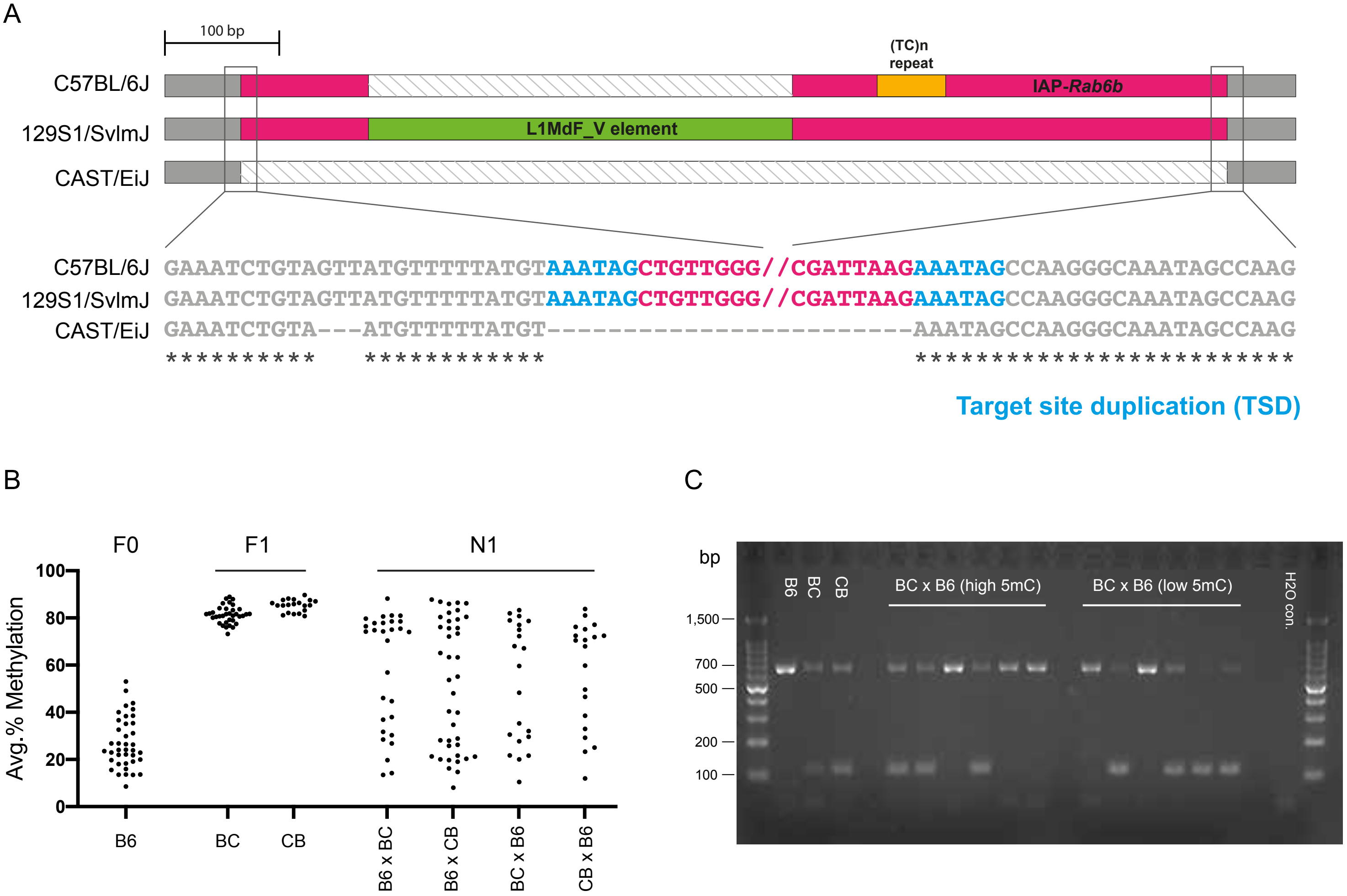

Fig. S3. Characterisation of IAP-*Rab6b* insertion site and inheritance patterns. (*A*) Alignment of the IAP-*Rab6b* insertion site in the B6, 129, and CAST genomes. DNA sequences were extracted from the GRCm38/mm10, 129S1_SvlmJ_v1, and CAST_EiJ_v1 genome assemblies accessed through the UCSC genome browser. IAP-*Rab6b* (pink) is absent in CAST mice and present in B6 and 129 mice. The B6 version of IAP-*Rab6b* contains a (TC)n repeat (orange) and the 129 version contains a LINE insertion of the L1MdF_V subclass (green). The bordering unique region and the target site duplication (TSD) sequences are shown in grey and blue, respectively. Asterisks indicate residue conservation. The majority of the IAP-*Rab6b* sequence is omitted (indicated with slashes). (*B*) Reacquisition of low methylation at IAP-*Rab6b* via genetic backcrossing regardless of breeding direction. Maternal genotype precedes paternal genotype for the listed N1 parental genotypes. Each dot represents the average DNA methylation level across CpGs for a single individual. (*C*) Segregation of IAP-*Rab6b* methylation states in N1 mice is independent of IAP-*Rab6b* copy number. Six highly- and six lowly methylated N1 individuals were genotyped via PCR using primers targeting the IAP-*Rab6b* flanking regions, amplifying a ~700bp B6 fragment containing IAP-*Rab6b* and a ~100bp CAST fragment lacking IAP-*Rab6b*. PCR product sizes were assessed via agarose gel electrophoresis. Since IAP-*Rab6b* is absent from the CAST genome, individuals exhibiting amplification of the larger fragment only are homozygous for IAP-*Rab6b* and individuals exhibiting amplification of both fragments are hemizygous for IAP-*Rab6b*. The B6, BC, and CB DNA samples are included as controls.

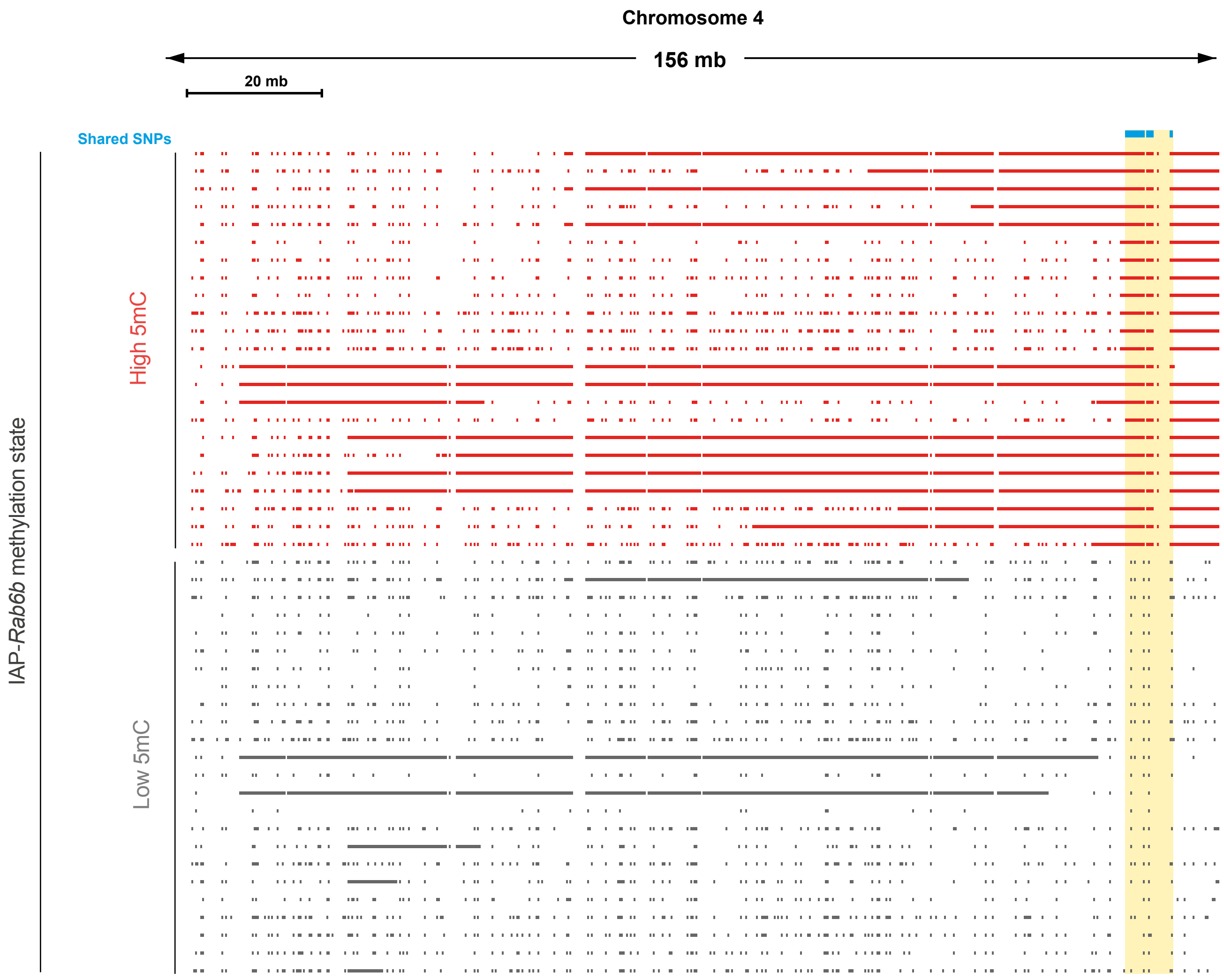

Fig. S4. Map of heterozygous SNPs along Chromosome 4 that are informative between B6 and CAST in N3 individuals. Heterozygous SNPs shared amongst all highly methylated N3 individuals (red) and absent from all lowly methylated N3 individuals (grey) are shown in blue. The corresponding mapped region is highlighted in yellow.

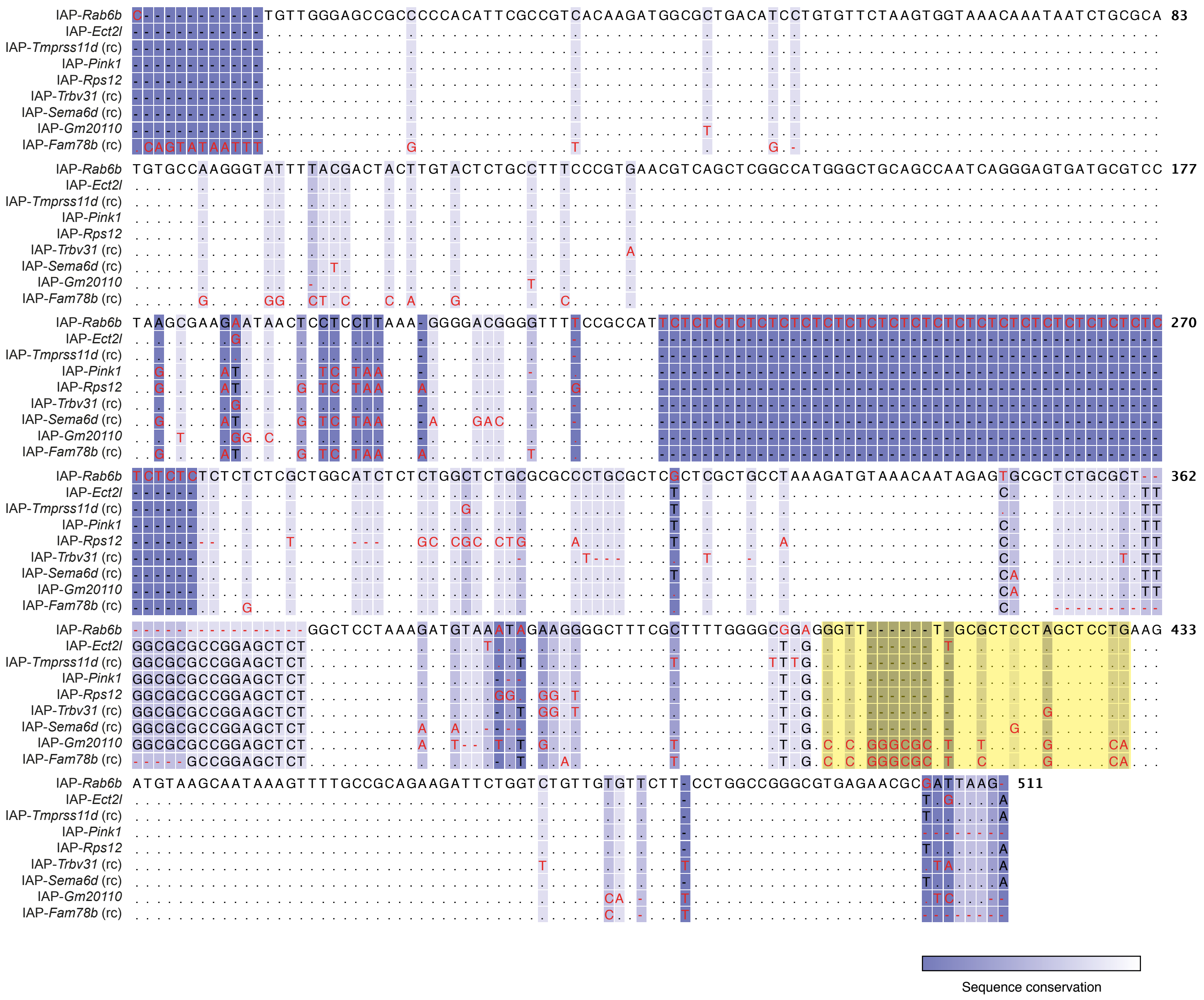

Fig. S5. Full-length sequence alignment of VM-IAPs identified as targets and non-targets of Chr4 cl. Sequences were extracted from the UCSC genome browser and contraoriented elements were reverse-complemented (rc) prior to generating the alignment. Dots represent conserved bases, dashes indicate lack of sequence, and divergent bases are shown in red. The 28 bp region displaying divergence between Chr4-cl targets and non-targets is highlighted in yellow.

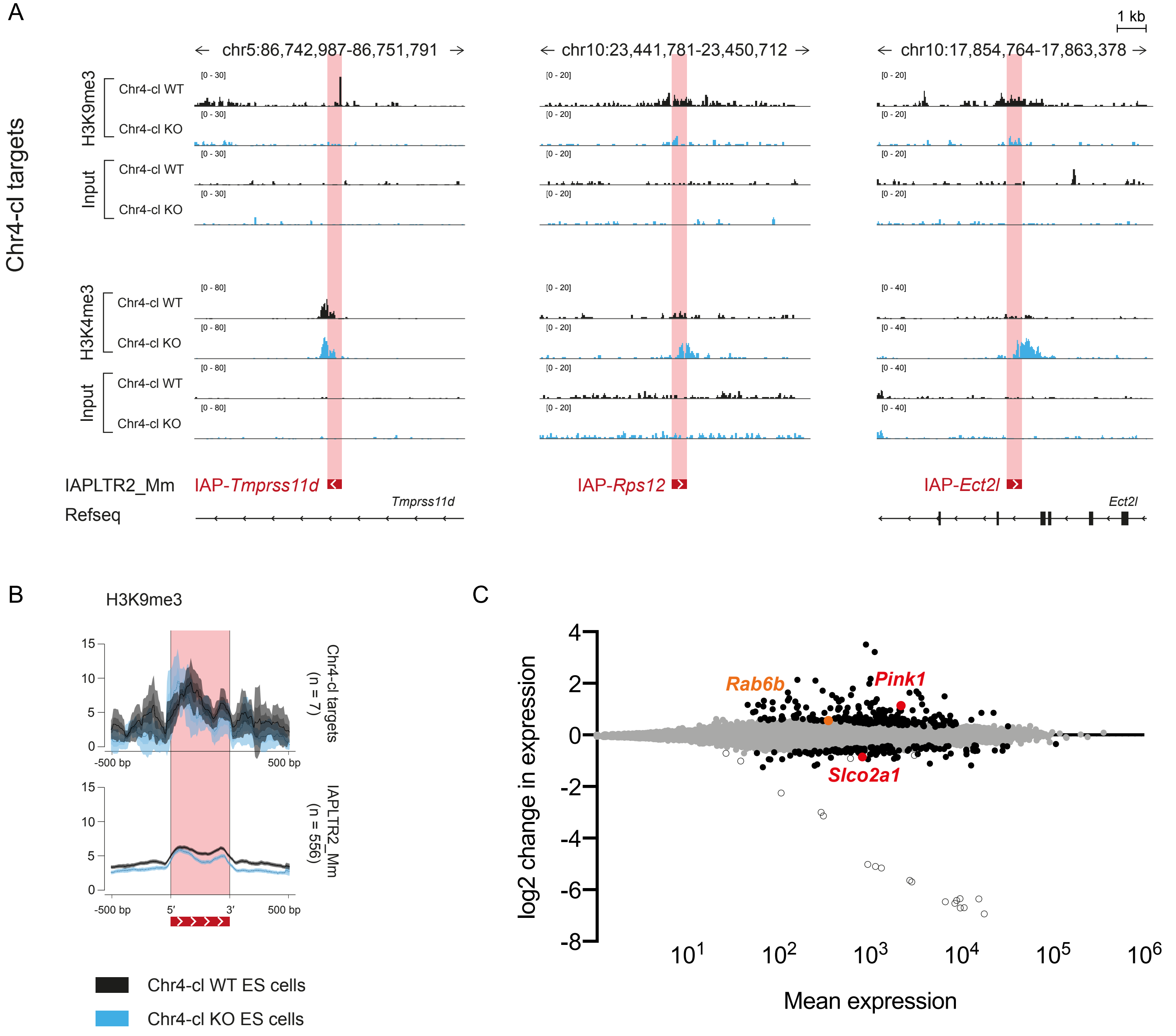

Fig. S6. Chromatin and transcriptional changes in Chr4-cl KO in ES cells. (*A*) H3K9me3 and H3K4me3 ChIP-seq signal at IAP-*Tmprss11d*, IAP-*Rps12*, and IAP-*Ect2l* in Chr4-cl WT (black) and KO (blue) ES cells of mixed B6/129 genetic background. VM-IAPs are shown in red and directionality is indicated with a white arrow. NCBI37/mm9 genome coordinates and neighbouring annotated genes are displayed above and below the ChIP-seq tracks, respectively. (*B*) Mean H3K9me3 ChIP-seq signal over the seven confirmed Chr4-cl targets (upper panel) and over all solo LTRs of the IAPLTR2_Mm subclass in the mouse genome (lower panel). Dotted lines represent mean signal and shaded regions represent error estimates (standard error and 95% confidence interval). Plots were generated using SeqPlots software (7). (*C*) Differential expression analysis between Chr4-cl WT and KO ES cells using RNA-seq data. Significantly up- and downregulated genes (adjusted p-value < 0.05) are shown in black.

Dataset Legends

Dataset S1 (separate file). Supplementary tables.

Table S1. Informative heterozygous GigaMUGA SNPs.
Table S2. Informative heterozygous MiniMUGA SNPs.
Table S3. IAP genomic coordinates.
Table S4. Primer sequences.
Table S5. Annotated Chr4-cl KZFPs.
Table S6. GEO accession numbers.

Dataset S2 (separate file). Unprocessed MiniMUGA SNP calls.

Dataset S3 (separate file). Unprocessed GigaMUGA SNP calls.

SI References

1. G. Wolf, *et al.*, Krab-zinc finger protein gene expansion in response to active retrotransposons in the murine lineage. *Elife* **9**, 1–22 (2020).
2. C. Nellåker, *et al.*, The genomic landscape shaped by selection on transposable elements across 18 mouse strains. *Genome Biol.* **13**, R45 (2012).
3. J. Lilue, *et al.*, Sixteen diverse laboratory mouse reference genomes define strain-specific haplotypes and novel functional loci. *Nat. Genet.* **50**, 1574–1583 (2018).
4. A. P. Morgan, *et al.*, The mouse universal genotyping array: From substrains to subspecies. *G3 Genes, Genomes, Genet.* **6**, 263–279 (2016).
5. H. Wickham, R. François, L. Henry, K. Müller, dplyr: A Grammar of Data Manipulation. https://dplyr.tidyverse.org/ (2019).
6. H. Thorvaldsdóttir, J. T. Robinson, J. P. Mesirov, Integrative Genomics Viewer (IGV): High-performance genomics data visualization and exploration. *Brief. Bioinform.* **14**, 178–192 (2013).
7. P. Stempor, J. Ahringer, SeqPlots - Interactive software for exploratory data analyses, pattern discovery and visualization in genomics. *Wellcome Open Res.* **1**, 14 (2016).
8. R. Hubley, *et al.*, The Dfam database of repetitive DNA families. *Nucleic Acids Res.* **44**, D81–D89 (2016).
9. T. L. Bailey, *et al.*, MEME Suite: Tools for motif discovery and searching. *Nucleic Acids Res.* **37**, W202–W208 (2009).
